# Measurement of Patient Satisfaction With the Trend of Virtual Clinics During the COVID-19 Pandemic

**DOI:** 10.7759/cureus.16016

**Published:** 2021-06-29

**Authors:** Afnan A Alwabili, Eman A Alotaibi, Ashwaq A AlE’ed, Ibrahim Alqunibut, Ola A Alotaibi

**Affiliations:** 1 Department of Medicine, Unaizah College of Medicine and Medical Sciences, Qassim University, Qassim, SAU; 2 Department of Family and Community Medicine, Unaizah College of Medicine and Medical Sciences, Qassim University, Qassim, SAU; 3 Department of Pediatrics, Unaizah College of Medicine and Medical Sciences, Qassim University, Qassim, SAU; 4 Department of Surgery, Unaizah College of Medicine and Medical Sciences, Qassim University, Qassim, SAU; 5 Department of General Pediatrics, Children's Specialized Hospital, King Fahad Medical City, Qassim, SAU

**Keywords:** covid-19, patient satisfaction, telemedicine, telehealth, virtual clinics

## Abstract

Background

The consequences of the coronavirus 2019 (COVID-19) pandemic on healthcare systems worldwide are undeniably disrupting regularly provided care for non-COVID-19 patients. Since the start of the pandemic, medical services in Saudi Arabia have adapted to the situation by providing medical care through virtual clinics. This article aims to evaluate patient satisfaction with virtual clinics during the COVID-19 pandemic.

Material and methods

A cross-sectional study was conducted among patients who had experience with the virtual clinics of Unaizah College of Medicine, Qassim University, Saudi Arabia. An online questionnaire was sent to all participants who visited the virtual clinic between May 2020 and July 2020. The questionnaire included demographic data and 16 statements to assess patient satisfaction with the virtual clinic experience.

Results

A total of 123 participants completed the questionnaire. Their mean age was 33 ± 12 years; 61% were females and 39% were males. Most of the participants were from the Qassim region (77.2%), while 22.8% were from other regions in Saudi Arabia. Dermatology clinics were the most frequently visited virtual clinics, followed by psychiatry clinics. Most of the participants were satisfied with the virtual clinics, with average scores greater than three for most of the components of the questionnaire.

Conclusions

The participants showed considerable satisfaction for virtual clinics in the time of the COVID-19 pandemic, even though the service was relatively new to them. Future additional efforts will be needed to support clinically appropriate and acceptable virtual visits combined with in-person visits after the pandemic.

## Introduction

The World Health Organization announced officially that the outbreak of coronavirus 2019 (COVID-19) cases had reached a pandemic level on March 11, 2020. Multiple action plans were developed in response to the pandemic; these included early identification, isolation of patients, symptomatic observation of contacts in addition to suspected and infected cases, and quarantines for public health [1]. Population isolation and the consequences of the pandemic on healthcare systems have affected the regularly provided care for non-COVID-19 patients. As a result, worldwide healthcare systems have increasingly relied on telemedicine, especially video consultation. The purpose is to facilitate the accessibility to health care and maintain the regular follow-up of outpatient care while minimizing the likelihood of virus spread within the community and hospitals [1].

The Saudi government and the ministry of health have recommended “social distancing” to reduce viral spread and have called for health systems to reduce non-urgent, in-person healthcare visits and planned elective procedures. To comply with these recommendations, the telehealth program in Saudi Arabia has expanded rapidly to provide the ability to sustain healthcare access during the pandemic [2].

Telehealth, also known as telemedicine, is characterized by the provision of distant healthcare services by utilizing modern technology for establishing diagnosis, direct management, injury and disease prevention, research and assessment, and healthcare provider education [3]. Telehealth has gained interest among clinicians and decision-makers, especially since the increased use of the internet since the late 1990s, with its potential opportunities for improving the cost and convenience of health care [4]. It is particularly useful for facilitating access to care and providing medical services at a distance when regular providers are not available [5]. Other benefits of telemedicine include overcoming travel costs, efficiency, cost-effectiveness, and high patient satisfaction [6]. A systematic review conducted in western Canada showed that telehealth has various advantages, including expanded access to facilities, enhanced care, and cost savings [7]. Other studies have indicated that telehealth facilities’ quality and the clinical outcome of cases during telehealth visits can be similar to conventional face-to-face visits with the added advantage of improved access to medical care [5].

For telehealth to be an applicable modality of delivering services, patient satisfaction is essential. Also, healthcare satisfaction is highly related to increased patient commitment and compliance with treatment for various conditions in multiple clinical settings [8,9]. A recent systematic review of the satisfaction of “patients and caregivers with telehealth videoconferencing” included 36 studies and showed high rates of satisfaction in the following dimensions: system experience, shared knowledge, user focus, and general satisfaction [10]. Prospective studies confirmed the safety, considerable patient satisfaction, and clinical equality in settings of both endourology and prostate cancer [11].

Another recent observational study on patients with colorectal cancer showed that video consultation is equivalent to in-person consultation regarding quality of acquired care and patient satisfaction [12]. In reviewing telemedicine studies in the Middle East, we found one study conducted in Jordan showing high patient satisfaction in tele-nephrology [13]. Another research project was carried out in Riyadh, Saudi Arabia, on the cost savings and satisfaction with telehealth among pediatric urology patients, which revealed an overall satisfaction rate of 89% [14].

There has been a growing demand for telehealth services in Saudi Arabia in the time of the COVID-19 pandemic. Thus, we initiated the virtual clinic concept to facilitate healthcare services. This study aimed to fill a knowledge gap in our region in regard to patient satisfaction with telehealth practice.

## Materials and methods

Study setting and subjects

This community-based cross-sectional descriptive study examined virtual clinics of Unaizah College of Medicine and Medical Science, Qassim University, Saudi Arabia. The study period was from May 5 to July 9, 2020. It is a newly established volunteering virtual clinics project not related to any previous physician’s clinic or hospital before the COVID-19 lockdowns. Therefore, it is eligibly accessible to any patient of any region or nationality who wishes to take advantage of the benefit by consulting a physician. The organization team consists of academic medical staff, project manager, secretory and technical experts of Unaizah College of Medicine and Medical Science. Eleven virtual clinics took part in the project. Their specialties included the following: internal medicine, family medicine, general surgery, pediatrics, psychiatry, adult neurology, dermatology, orthopedics, obstetrics and gynecology, otolaryngology, ophthalmology, public health, and preventive medicine.

The software utilized in this project is Zoom®, in which the patients and physicians communicated via two-way audio and visual means using video monitors. Under the guidance of technical experts, each physician generates its private room with the Zoom® software. We ensure that each patient is seen separately through technical properties available in the program, as confidentiality is one of our important concerns. 

The administrative secretary organized the appointment request in which each patient filled out an online consultation request form and scheduled an appointment to be held within one week. Then the patients received a video administration and instruction of using the virtual clinic with provided technical support contact prior to their appointment. At the end of the consultation session, the patients were informed of the study and its purpose; those who gave informed consent received an online questionnaire to evaluate their satisfaction level.

Study subjects

We included in our study all participants who logged in to virtual clinics and agreed to be part of the research. The study subjects were Saudis and non-Saudis of all age groups and included both males and females. Those with considerable communication difficulties, including severely impaired hearing or vision, severe neurocognitive disorder, or a mental condition leading to non-communication, were excluded.

Data collection

Data was systemically retrieved using a structured questionnaire built by study investigators in keeping with its objectives and literature review of related studies [12,15]. This approach was made to cover all aspects that needed to be addressed in regards to this topic. The questionnaire’s first section explored the demography and basic background of our study participants. The second section contained 16 items to evaluate the satisfaction of patients with virtual clinic experiences.

These 16 items were classified into four categories: technical aspects, quality of acquired care, administrative aspects, and overall impression of virtual clinics. Each item was scored on a five-point Likert scale ranging from strongly disagree to strongly agree. The items were analyzed separately. The questionnaire was thoroughly reviewed by two research experts and tested on a small sample of 20 participants before being finalized. Those who were enrolled in the pilot were excluded from the final analysis.

Data analysis

The analysis of data was performed using the Statistical Package for Social Sciences (SPSS), version 24 (IBM Corp., Armonk, NY, USA). The percentages represented the descriptive statistics for categorical variables, while continuous variables were expressed using standard deviation and mean. The χ2 test was applied to evaluate differences between categorical variables in analytical statistics. An independent Student’s t-test was utilized to compare means. The level of statistical significance was at p ≤ 0.05.

## Results

The questionnaire was filled out by 123 participants. Table 1 provides an overview of the study participants’ demography and basic backgrounds. The mean age was 33 ± 12 years; females made up 61% of the study population while 39% were male. Most participants were married (60.2%) and had educational levels of college degrees or higher (68.3%). They were mainly from the Qassim region (77.2%), with the remaining 22.8% from other areas of Saudi Arabia.

**Figure 1:**
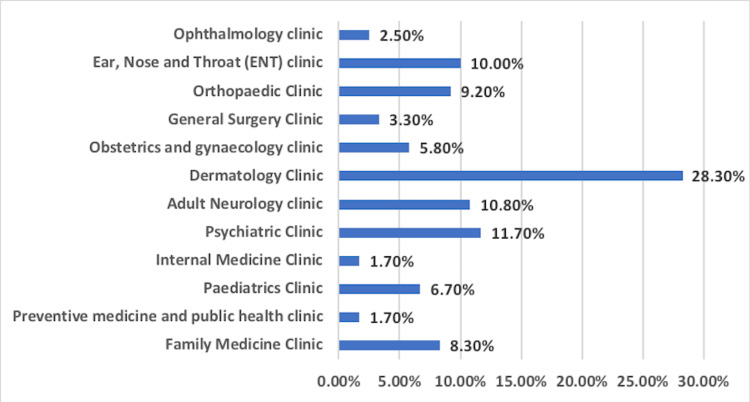
Distribution of virtual clinic visit across different medical specialties

**Figure 2:**
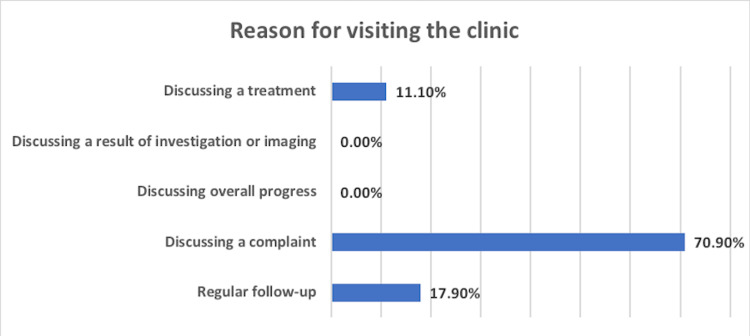
Reasons for visiting the virtual clinic

Dermatology clinics were visited the most (28.3%), followed by the psychiatric clinic (11.7%), neurology clinic (10.8%), and ear, nose, and throat (ENT) clinic (10%). Other clinics were visited with minor frequency (ranging from 1.7% for preventive medicine to 9.2% for orthopedic clinics) (Figure 1). The reason behind the visits was most commonly to discuss a complaint (70.9%), followed by regular follow-up (17.9%) and discussing a treatment regimen (11.1%) (Figure 2). Furthermore, 94.3% of the study population had never visited a virtual clinic before, while only 5.7% had visited virtual clinics previously.

**Table 1:** Participants’ demographics and background characteristics

Characteristic		Count	Total %
Age (Mean = 33 ± 12) & Gender:	Male	48	39.0%
	Female	75	61.0%
Marital status:	Single	46	37.4%
	Married	74	60.2%
	Divorced	3	2.4%
Educational level:	Illiterate	2	1.6%
	Elementary	3	2.4%
	Junior high	19	15.4%
	Secondary	2	1.6%
	Diploma	13	10.6%
	College and higher	84	68.3%
Region:	Qassim region	95	77.2%
	Outside the Qassim region	28	22.8%
Have you ever tried virtual clinics before?	No	116	94.3%
	Yes	7	5.7%

To determine the satisfaction level of participants toward virtual clinics, we measured several dimensions, as shown in Table 2. There was a high satisfaction level toward technical aspects with a mean of 4.53 out of 5. There was also a high satisfaction level toward the quality of acquired care with a mean of 4.73 out of 5. The overall satisfaction toward administrative aspects was high with a mean of 4.37 out of 5, and the impression of virtual clinics was highly positive with a mean of 4.28 out of 5. Accordingly, the overall satisfaction level toward all dimensions was 4.49 out of 5.

**Table 2:** Patient satisfaction toward the virtual clinic

	Strongly disagree	Disagree	Neutral	Agree	Strongly agree		
	Count (%)	Count (%)	Count (%)	Count (%)	Count (%)	Mean	STD*
Technical Aspects							
I can see my doctor clearly	3 (2.4)	5 (4.1)	18 (14.6)	21 (17.1)	76 (61.8)	4.32	1.027
I can hear my doctor clearly	3 (2.4)	0 (0.0)	3 (2.4)	15 (12.2)	102 (82.9)	4.73	.736
There is no significant lag between sound and video	4 (3.3)	2 (1.6)	12 (9.8)	24 (19.5)	81 (65.9)	4.43	.967
I am overall satisfied with the technical aspects of using the virtual clinic	0 (0.0)	0 (0.0)	7 (5.7)	28 (22.8)	88 (71.5)	4.66	.584
Overall Technical Aspects						4.5346	.61598
Perceived Quality of Care Received							
I feel relaxed while interacting with my physician	2 (1.6)	0 (0.0)	1 (0.8)	21 (17.1)	99 (80.5)	4.75	.635
I feel comfortable discussing and addressing all of my concerns	1 (0.8)	1 (0.8)	3 (2.4)	19 (15.4)	99 (80.5)	4.74	.625
I feel understood by my physician during the virtual clinic visit	1 (0.8)	1 (0.8)	2 (1.6)	17 (13.8)	102 (82.9)	4.77	.598
I feel that there was enough time to address my concerns	1 (0.8)	2 (1.6)	4 (3.3)	20 (16.3)	96 (78.0)	4.69	.691
I feel that my privacy was respected	3 (2.4)	1 (0.8)	0 (0.0)	20 (16.3)	99 (80.5)	4.72	.741
Overall Perceived Quality of Care Received						4.7333	.53755
Administrative Aspects							
The appointment was scheduled easily	0 (0.0)	0 (0.0)	2 (1.6)	22 (17.9)	99 (80.5)	4.79	.448
I know what to expect during virtual clinic visits	4 (3.3)	13 (10.6)	18 (14.6)	36 (29.3)	52 (42.3)	3.97	1.138
Overall Administrative Aspects						4.3780	.63485
Impression of virtual clinics							
I would use virtual clinics again	1 (0.8)	0 (0.0)	1 (0.8)	21 (17.1)	100 (81.3)	4.78	.536
It is easier for me to see my healthcare provider via virtual clinic than face to face	1 (0.8)	2 (1.6)	12 (9.8)	20 (16.3)	88 (71.5)	4.56	.801
I prefer seeing my physician in person rather than via virtual clinic	8 (6.5)	25 (20.3)	43 (35.0)	18 (14.6)	29 (23.6)	3.28	1.218
In the future, I would like to use a combination of virtual clinics and face-to-face visits	4 (3.3)	1 (0.8)	12 (9.8)	32 (26.0)	74 (60.2)	4.39	.938
A virtual clinic makes me less dependent on others	3 (2.4)	1 (0.8)	14 (11.4)	29 (23.6)	76 (61.8)	4.41	.905
Overall Impression of Virtual Clinics						4.2862	.46170
Overall Satisfaction						4.4995	.40159

Table 3 shows a statistically significant relationship between satisfaction level toward most dimensions and the type of clinic visited (P-value < 0.05) except between the dimensions of administrative aspects and impression toward the clinic (P-value > 0.05). Participants who visited dermatology clinics were most satisfied with technical aspects. Participants who visited preventive medicine and public health clinics were most satisfied with perceived quality. Overall satisfaction was highest among participants who visited the family medicine clinic.

**Table 3:** Relationship between patient satisfaction and type of virtual clinic visited * Technical Aspects, ** Perceived Quality of Care, *** Administrative aspect

	The type of virtual clinic visited	N	Mean Rank	Chi-Square	p-value
Overall TA*	Family Medicine Clinic	10	75.00	33.097	.001
	Preventive Medicine and Public Health Clinic	2	23.50		
	Pediatric Clinic	8	66.06		
	Internal Medicine Clinic	2	44.50		
	Psychiatric Clinic	14	43.82		
	Adult Neurology Clinic	13	45.23		
	Dermatology Clinic	34	82.19		
	Obstetrics and Gynecology Clinic	7	62.64		
	General Surgery Clinic	4	59.75		
	Orthopedic Clinic	11	43.27		
	Otolaryngology Clinic	12	47.67		
	Ophthalmology Clinic	3	41.33		
Overall PQC**	Family Medicine Clinic	10	74.40	26.616	.005
	Preventive Medicine and Public Health Clinic	2	81.50		
	Pediatric Clinic	8	75.06		
	Internal Medicine Clinic	2	55.75		
	Psychiatric Clinic	14	35.89		
	Adult Neurology Clinic	13	38.54		
	Dermatology Clinic	34	64.65		
	Obstetrics and Gynecology Clinic	7	75.21		
	General Surgery Clinic	4	54.50		
	Orthopedic Clinic	11	70.36		
	Otolaryngology Clinic	12	64.75		
	Ophthalmology Clinic	3	48.00		
Overall AA***	Family Medicine Clinic	10	73.85	9.400	.585
	Preventive Medicine and Public Health Clinic	2	60.00		
	Pediatrics Clinic	8	69.44		
	Internal Medicine Clinic	2	48.25		
	Psychiatric Clinic	14	50.54		
	Adult Neurology Clinic	13	56.27		
	Dermatology Clinic	34	68.15		
	Obstetrics and Gynecology Clinic	7	58.86		
	General Surgery Clinic	4	51.88		
	Orthopedic Clinic	11	62.41		
	Otolaryngology Clinic	12	43.04		
	Ophthalmology Clinic	3	57.00		
Overall Impression	Family Medicine Clinic	10	76.40	9.092	.613
	Preventive Medicine and Public Health Clinic	2	49.00		
	Pediatric Clinic	8	58.00		
	Internal Medicine Clinic	2	32.00		
	Psychiatric Clinic	14	55.14		
	Adult Neurology Clinic	13	52.96		
	Dermatology Clinic	34	66.51		
	Obstetrics and Gynecology Clinic	7	61.57		
	General Surgery Clinic	4	35.75		
	Orthopedic Clinic	11	62.14		
	Otolaryngology Clinic	12	54.58		
	Ophthalmology Clinic	3	78.50		
Overall Satisfaction	Family Medicine Clinic	10	85.70	26.955	.005
	Preventive Medicine and Public Health Clinic	2	39.00		
	Pediatrics Clinic	8	72.56		
	Internal Medicine Clinic	2	45.00		
	Psychiatric Clinic	14	36.89		
	Adult Neurology Clinic	13	42.92		
	Dermatology Clinic	34	75.88		
	Obstetrics and Gynecology Clinic	7	67.21		
	General Surgery Clinic	4	50.88		
	Orthopedic Clinic	11	56.18		
	Otolaryngology Clinic	12	48.50		
	Ophthalmology Clinic	3	42.00		

Table 4 shows no statistically significant relationship between satisfaction level toward most dimensions and the reason for visiting the virtual clinics (P-value > 0.05). However, there was an exception between the dimensions of administrative aspects, impression toward use, and overall satisfaction (P-value = 0.009, 0.057, and 0.004, respectively). The participants who visited the clinics for regular follow-up were the most satisfied.

**Table 4:** Relationship between patient satisfaction and reason for visiting the virtual clinics * Technical Aspects, ** Perceived Quality of Care, *** Administrative aspect

	Reason for visiting the clinic:	N	Mean Rank	Chi-Square	p-value
Overall TA*	Regular follow-up	21	70.40	4.540	.103
	Discussing a complaint	83	55.10		
	Discussing a treatment	13	65.46		
Overall PQC**	Regular follow-up	21	63.10	.581	.748
	Discussing a complaint	83	58.42		
	Discussing a treatment	13	56.08		
Overall AA***	Regular follow-up	21	78.55	9.521	.009
	Discussing a complaint	83	55.19		
	Discussing a treatment	13	51.77		
Overall Impression	Regular follow-up	21	73.71	5.716	.057
	Discussing a complaint	83	54.60		
	Discussing a treatment	13	63.35		
Overall Satisfaction	Regular follow-up	21	80.31	10.922	.004
	Discussing a complaint	83	53.15		
	Discussing a treatment	13	61.92		

The correlation between satisfaction level and participants’ demographics, including gender, marital status, residency, and education, was not significant statistically. Furthermore, there was no statistically significant relationship between satisfaction level toward all dimensions and whether the participants had previously visited the virtual clinics or not. 

## Discussion

The utilization of telemedicine was sluggish before COVID-19, but the emergence of pandemic circumstances is exceptional, necessitating an urgent reorganization of practices to limit the transmission of infection. Numerous face-to-face medical visits have been barred as a result of the pandemic’s effects. This closure has led to disruption of the regularly provided care and makes patients reluctant to seek medical consultations [16]. To cope with this, various medical services have changed to be delivered via virtual visits [17]. Telemedicine consultation is helping to link the gap between patients and providers during this pandemic by allowing them to interact through virtual services while remaining at home and making follow-up possible [18].

Within a matter of weeks, our project was able to set up and assigned multiple clinical specialties in the virtual clinics, although no resources were available before the virus outbreak. The goal was to maintain care continuity without putting patients or physicians at risk, as well as to prevent unnecessary contact during transit to the hospital and in waiting rooms. Our project was capable of facilitating healthcare service among participants from the Qassim region and outside the region by 77.2% and 22.8%, respectively. This highlights the important role of telemedicine in providing medical care by various specialized physicians when regular providers are unavailable in other remote areas.

The virtual visit was equally accessible across all physicians from different subspecialty groups. Moreover, the psychiatry and dermatology clinics notably recorded the highest number of visits to a virtual clinic at 28.3% and 11.7%, respectively. We did not explicitly investigate the grounds for this disparity, but it may be related to the differences in how each specialty approaches its patients [19,20].

One explanation for this is that the dermatologist can use online means to accurately assess a patient’s condition and skin lesions through video calls and live photographs without the patient being present at the clinic [21]. Regarding telepsychiatry, mental health is one of the most rapidly growing fields in recent years [22], and many psychological issues can arise secondary to the virus pandemic and quarantine [23]. Also, we observed that the virtual mental health clinic circumvents concerns regarding confidentiality and stigma associated with using in-person services of mental health.

Most of our participants (94.3%) had no previous experience with virtual clinics. This indicates the important role of this project in raising awareness about telemedicine as a means to provide healthcare services. Even though most of our participants had never used virtual clinics before, a high percentage of them were satisfied with the virtual clinics, with average scores greater than three for most of the questionnaire’s components. This finding is in line with previous research on telemedicine patient satisfaction within the COVID-19 period [24].

One of the most critical aspects of conducting virtual clinics is technical quality. More than 90% of our participants were satisfied with the visio-audio quality. This contrasts with previous research, which showed that patients were unable to hear the clinician clearly during the virtual visit [25]. In another study, video quality was rated quite low [26].

While it might appear at first that a virtual visit offers less personal contact and acts as a barrier to developing a doctor-patient relationship, this did not appear to be the case in our research. Physicians could maintain empathy via verbal expressions with nonverbal communication by utilizing a simultaneous audiovisual network through virtual clinics. In our results, over 80% of the study population reported that the physicians addressed and understood their concerns, and they desired to engage in future virtual visits. Furthermore, 85% of the responses indicated a preference for using a combination of virtual clinics and face-to-face visits in the future. This combination will facilitate care continuity and provide adequate follow-up visits when compared with in-person visits only. Several studies found that even after the COVID-19 pandemic, healthcare practitioners and patients have expressed an interest in using telemedicine as a portion of their regular follow-up visits [27-29].

A simple in-person appointment may require considerable preparation and economic cost for people who rely on caregivers or public transport to get to appointments. Allowing these people to communicate with their physicians from home through virtual channels would make it easier for them to receive healthcare services without relying on others [30]. This is similar to our finding that 85% of participants became less dependent on others after using the virtual clinics.

While telemedicine is currently required for social distancing to minimize the exposure risk of COVID-19, we hypothesize that patients will continue to favor telemedicine in the near future due to its potential to save time and improve access to specialty care. These advantages must be balanced against the benefits of an in-person assessment, which include a more personable interaction and the capability to perform a physical examination or diagnostic testing. We believe that the combination of telemedicine and in-person assessment within the health system will encourage providers and patients to obtain the advantages of both.

Although virtual clinic adoption became trendy in multiple settings during the pandemic of COVID-19 [24], to our knowledge, this is the first study of rapidly established virtual clinics including all medical specialties in the Qassim region through an evaluation of patient satisfaction. However, this study had some limitations that need to be recognized. The lack of follow-up is one of the study’s limitations. However, our study’s goal was to address the current patient satisfaction with telehealth practice. Also, the results may not be generalizable to other areas of the country where telemedicine is unavailable. In addition, due to the short study duration, the sample size was small. Finally, a comparable in-person control group was inaccessible due to the social distancing barrier.

## Conclusions

During the pandemic of COVID-19, telemedicine has become a prominent solution for healthcare delivery and continuity, with a possible impact on clinical practice post-pandemic. The study's findings demonstrate that virtual consultations can be rapidly instituted in adaptation to a coronavirus 19 sequelae with a high level of patient satisfaction and willingness of future engagement in the virtual clinic despite being a relatively new service and experience to the participants. After the pandemic, additional efforts will be needed to support clinically appropriate and acceptable virtual visits combined with in-person visits to be established in the Qassim region's health services.
